# Effects of Probiotics and Synbiotics on Weight Loss in Subjects with Overweight or Obesity: A Systematic Review

**DOI:** 10.3390/nu13103627

**Published:** 2021-10-17

**Authors:** Valentina Álvarez-Arraño, Sandra Martín-Peláez

**Affiliations:** 1 Departamento de Medicina Preventiva y Salud Pública, Facultad de Medicina, Universidad de Granada, 18071 Granada, Spain; vpalvarez@correo.ugr.es; 2 Instituto de Investigación Biosanitaria de Granada, 18012 Granada, Spain

**Keywords:** obesity, overweight, weight loss, probiotics, synbiotics, microbiota

## Abstract

Intestinal microbiota has been shown to be a potential determining factor in the development of obesity. The objective of this systematic review is to collect and learn, based on the latest available evidence, the effect of the use of probiotics and synbiotics in randomized clinical trials on weight loss in people with overweight and obesity. A search for articles was carried out in PubMed, Web of science and Scopus until September 2021, using search strategies that included the terms “obesity”, “overweight”, “probiotic”, “synbiotic”, “*Lactobacillus*”, “*Bifidobacterium*” and “weight loss”. Of the 185 articles found, only 27 complied with the selection criteria and were analyzed in the review, of which 23 observed positive effects on weight loss. The intake of probiotics or synbiotics could lead to significant weight reductions, either maintaining habitual lifestyle habits or in combination with energy restriction and/or increased physical activity for an average of 12 weeks. Specific strains belonging to the genus *Lactobacillus* and *Bifidobacterium* were the most used and those that showed the best results in reducing body weight. Both probiotics and synbiotics have the potential to help in weight loss in overweight and obese populations.

## 1. Introduction

The Western Diet, characterized by a large consumption of processed products, saturated fats, sugars and a low fiber content, together with an increasingly sedentary lifestyle, has generated tripled levels of obesity in the world as compared to the year 1975, according to the World Health Organization [1]. In fact, obesity is currently classified as an epidemic and has become one of the greatest public health challenges in the twenty-first century [2].

Obesity is defined as an excessive accumulation of fat and hypertrophy of adipose tissue [1]. It is a chronic pathology, where the fundamental cause is the imbalance between the calories consumed and those expended [3]. However, it is considered a multi-causal and complex disease influenced by factors intrinsic and extrinsic to the individual, such as environmental, genetic, neuronal, endocrine and behavioral components [3,4]. Furthermore, overweight and obesity are risk factors for other chronic diseases, such as diabetes *mellitus* II, cardiovascular diseases and some types of cancer [3].

It has recently been shown that obesity and its association with other chronic non-communicable diseases are not only the result of genetic factors, eating habits or lack of physical activity; it has also been proven that the Intestinal Microbiota (IM) is an environmental factor in its development [5,6,7,8].

People with overweight or obesity have been shown to have a specific IM profile, characterized by dysbiosis (imbalance) and lower microbial diversity compared to people with normal weight [6,9]. In this sense, a decrease has been seen in some bacterial phyla, such as the relationship between *Bacteroidetes/Firmicutes* [10,11], with lower proportions of *Bacteroidetes* and higher proportions of *Firmicutes* than those from people without obesity [12]. This seems to facilitate energy extraction from the ingested food and increases energy storage in the host’s adipose tissue [7]. In addition, this altered microbiota also results in a suppressed production of fasting-induced adipose factor (Fiaf). This suppression leads to an increased storage of triglycerides in adipose tissue and a low release of hormones such as glucagon-like peptide 1 (GLP-1) and the peptide YY (PYY), promoting food intake. Although research in humans is incipient, there are clear indications that the intestinal microbiota is important for maintaining homeostasis of energy metabolism [13,14,15].

The International Scientific Association of Probiotics and Prebiotics (ISAPP) defines probiotics as “live microorganisms that, after ingestion in specific numbers, exert benefits for the health of the host” [16] and prebiotic as “a substrate that is selectively utilized by host microorganisms conferring a health benefit” [17]. A synbiotic, is defined as “a mixture comprising live microorganisms and substrate(s) used selectively by host microorganisms that confers a benefit to the health of the host” [18].

This systematic review evaluates the effect of probiotic and synbiotic intake on IM in reducing body weight and/or body fat in apparently healthy people with overweight or obesity in randomized clinical trials.

## 2. Materials and Methods

This systematic review was reported according to the PRISMA statement [19].

### 2.1. Search Strategy

In order to address the proposed objective, a systematic review has been carried out, finishing in September 2021 in three health science databases (Pubmed, Web of Science and Scopus).

Searching strategies were constructed using controlled language from the Medical Subject Headlines (MeSH) (Probiotics, *Lactobacillus*, Synbiotics, Overweight, Obesity, Weight Loss) and health science descriptors (DeCs) (Probiotic *, *Lactobacillus*, Synbiotic *, Overweight, Obesity, Weight loss, Bifidobacteria). Additionally, to define the union between the terminologies, the Boolean operators “AND”, “OR” and “NOT” were used. The truncation (*) was also used in order to encompass all words related to probiotic, and synbiotic.

To further delimit the results, additional filters were applied to each database. The specific details of the aforementioned strategies are given in Table S1.

### 2.2. Eligibility Criteria

The selection criterion were randomized clinical trials carried out in humans, published in the last 10 years, in apparently healthy people classified as overweight or obese, according to Body Mass Index (BMI; overweight 25–29.9 kg/m^2^, obese ≥ 30 kg/m^2^), body fat percentage (females: overweight 26.0–31.9%, obese ≥ 32%; males: overweight 21.0–24.9%, obese ≥ 25%), visceral fat area or waist circumference (obese females ≥ 80 cm, obese males ≥ 94 cm) [20,21] in all age groups, where it was evaluated the effect of taking a probiotic or synbiotic on weight loss. The published results of the study required to be written in English. Review articles, studies of people with other chronic non-communicable diseases, carried out on animals, in pregnant participants or in the breastfeeding stage were excluded. The selection process can be found in Section 3.1.

### 2.3. Data Extraction and Analysis

Data was extracted by one of the authors (VA-A) and contrasted with the other co- author (SM-P). From each selected publication, information about the name of the main author, year and place where the study was carried out, population characteristics, study design, intervention, comparison, strains and doses used, period of intervention and main results were extracted.

### 2.4. Quality Assessment

To assess the quality of the 27 studies included in the systematic review, the Jadad scale [22] was used. It consists of 5 questions related to methodological quality. The following criteria are evaluated: whether the trial is randomized and double blind, whether a description of exclusions and dropouts is detailed, and finally if the randomization and double blind method are adequate. A score of 5 points correspond to the maximum quality level, whereas a score < 3 points is considered to indicate poor quality.

## 3. Results

### 3.1. Selection Process

**Figure 1:**
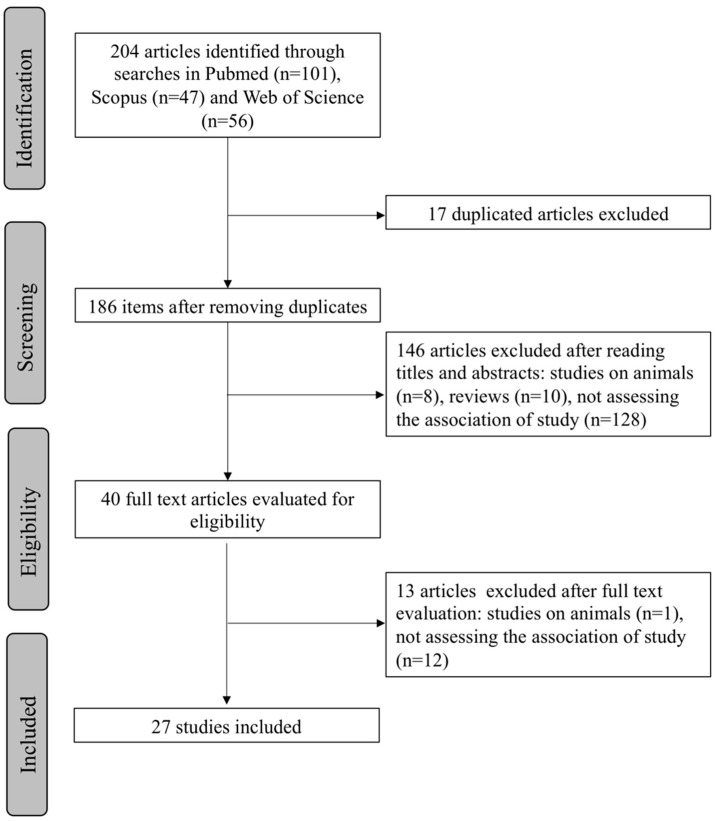
PRISMA flowchart showing the process of article selection.

Based on the search carried out, a total of 204 articles (101 Pubmed, 47 Scopus, 56 Web of Science) were obtained using the strategy previously described. From the initial search results, 17 duplicate articles were discarded. After screening of the titles and abstracts of the remaining studies, 146 articles not meeting the eligibility criteria (8 studies carried out on animals, 10 reviews, 128 studies not assessing the association of our study) were excluded. From the 40 articles that were read in their entirety, 13 did not meet the selection criteria (1 study carried out on animals, 12 studies not assessing the association of our study) and were also discarded. Finally, 27 articles were included in this systematic review. A flow chart illustrating the selection process is presented in Figure 1.

### 3.2. Study Characteristics

Table 1 presents the collection of the selected studies in an orderly and summarized manner, including the country, author or authors, methodological design, intervention and a summary of the main results.

The articles came from different countries, the majority being from Asia, where the most prevalent countries were Iran, South Korea and Japan. The second most prevalent countries were from the European continent, including Spain [39,42], Bulgaria [43,48] and Turkey [31]. The remaining countries were the United States [47], Canada [24,25] and Brazil [35]. From the 27 selected studies, 24 were conducted in adult populations and three in children [26,31,37]. Most of the studies considered both sexes, with the exceptions of [29,35,44,50], which were conducted in female only. The average age of the people included in the trials was 31.1 years, and the average BMI was 30.5 kg/m^2^.

Regarding the typology of the selected articles, they all had a quantitative approach, being randomized controlled clinical trials. Although most of the studies were double-blind, we found also single [31,42,44,47,50] and triple blind [26] studies. The most of the selected articles used probiotics except seven studies which investigated the effect of synbiotics [26,31,37,41,42,47,51]. Intervention duration average was 12 weeks, being 1 week the minimum [31] and 36 weeks the maximum [48] duration. Finally, the minimum sample size included 20 subjects [47] and the maximum, 225 [43].

Regarding the probiotic bacteria used in both probiotics and synbiotics, the genus *Lactobacillus* standed out, which included strains from the species *L. rhamnosus*, *L. gasseri*, *L. plantarum*, *L. casei*, *L. lactis*, *L. acidophilus*, *L. delbrueckii*, *L. reuteri* and *L. curvatus*. Regarding *Bifidobacterium*, it was common the use of strains belonging to the species *B. animalis*, *B. bifidum*, *B. lactis* and *B. breve*.

Probiotics and synbiotics were administered mainly through capsules, but also powders [30,31,34,49] and food products, mainly fermented dairy products such as probiotic yogurts [24,34,36,50] or fermented milks [27].

From the 27 studies, 11 combined the intervention with probiotics/synbiotics with other weight-loos strategies [25,28,29,36,37,38,42,44,47,48,50]. These combinations were successful in all of them but in two [33,47], which did not find significant differences. From the studies using either probiotics or synbiotics as unique intervention, only one did not find significant effects [24].

### 3.3. Probiotic Strains, Daily Doses and Total Intervention Doses

Most of the studies using fermented foods for the intake of probiotics contained lactic acid bacteria (LAB) as starter cultures, coming from the genus *Lactobacillus* and *Bifidobacterium* mainly.

On the other hand, we found a great diversity in terms of probiotic species and strains used to treat overweight and/or obesity. Most of the studies reported the probiotic/symbiotic formulations at the strain level, either using multi-strains [29,30,33,40,44,45,47,48,52] or, single- strain [23,25,27,34,36,38,39,46,49,51] in their formulations.

When used as single-strain, all probiotic interventions showed positive effects in decreasing body weight, BMI, waist circumference, body fat mass or fat percentage. These strains belonged to the genera *Lactobacillus* (*L. rhamnosus* CGMCC1.3724 (LPR) [25], *L. gasseri* BNR17 [38], *L.gasseri* SBT2055 [27], *L. sakei* CJLS03 [46] and *L. plantarum* Dad-13 [49]), *Bifidobacterium* (*B. lactis* Bb-12 [36], *B. animalis* ssp. *Lactis* 420 (B420) [51], *B. animalis CECT8145* [39]) and *Pediococcus* (*Pediococcus pentosaceus* LP28 [34]).

The multi-strain combinations were multiple and are detailed in Table 1.

The probiotic amount and duration of the intervention studies varied, from a maximum dose of 5 × 10^10^ [19,20] and the minimum dose of 1 × 10^6^ [24], and from 4 [21] to 36 [20] weeks, respectively.

### 3.4. Quality Assessment

Table S2 shows the evaluation of the methodological quality of the 27 randomized clinical trials included in this systematic review, carried out using the Jadad scale. A total of seven studies were rated with poor methodological quality [31,37,42,44,46,47,50] (<3 points), and six studies obtained an acceptable score [23,24,26,27,28,29,36] (3 or 4 points). The remaining studies obtained the maximum score (5 points). Most of the studies clearly described the method of randomization used in the study, as well as the blinding procedure. Others, however, did not explain the method of allocation concealment or blinding, and some were not defined as double-blind randomized trials, thus scoring lower.

## 4. Discussion

In recent years, the effect of pre- and probiotics has gained a great deal of interest in the treatment of overweight and obesity. The results of this systematic review indicate that probiotics and synbiotics, whether used as single-strain or multi-strain, could have a favorable effect on weight loss and other related anthropometric markers in people with overweight or obesity. In particular *L. gasseri* [23,27], different strains of *L. acidophilus* alone or together with different strains of the genera *Bifidobacterium* [31,43,48] or *Lactobacillus* [30,40] showed reducing effects even when the participants did not undergo energy restriction; however these interventions had in common an intervention period ≥ 8 weeks.

Trials finding positive results also at anthropometric level combined part or all of the intervention with a hypocaloric diet, calorie restriction, and/or increased physical activity. Consequently, weight loss was not assigned solely to the effect of the probiotic. Therefore, the real results of the strain(s) used in these trials are somewhat biased, especially in those trials where the interventions were very short (4 weeks) [21].

Studies with *L. gasseri* showed a decrease in body weight [27], BMI [23,27], waist circumference [23,27,38] and areas of visceral [23,27] and subcutaneous fat [23]. High body mass has been reported to be strongly associated with risk factors for cardiovascular diseases in both childhood and adulthood [53,54]. *L. gasseri* BNR17 was associated with a decrease in visceral adipose tissue in waist and hip circumferences post-consumption [38]. In another trial carried out in adults with diabetes with the same strain, only a slight reduction was observed (without being significant) in body weight and waist circumference; a result possibly associated with the sample size [55]. Kadooka et al. [27] used *L. gasseri* SBT2055 and observed a decrease in visceral fat area, BMI and waist and hip circumference at doses of 10^6^ CFU/g. When the same authors [23] used the same probiotic strain in a higher dose, observed also a reduction in the abdominal subcutaneous fat area, which suggests that at lower doses this strain can reduce its effectiveness.

The probiotics *L. curvatus* HY7601 with *L. plantarum* KY1032 showed reductions in body weight, BMI and waist circumference [30], what reinforces previous results carried out in mice, where they showed a reduction in weight gain and accumulation of fat, through the modulation of the intestinal microbiota [56].

*L. acidophilus* in combination with *L. casei* and *Bifidobacterium*, maintaining the usual lifestyle of the participants, showed positive effects in reducing body weight [41]. Another study using *L. acidophilus* with *B. infantis* obtained the same results [57]. Some *Lactobacillus* species, including *L. acidophilus*, have been associated with weight gain, due to their limited ability to break down fructose or glucose [58]. However, some species of the genus *Lactobacillus* in combination with other probiotics seem to favor weight loss [29,30,31,36,37,38,40,41,42,43,44,45,48,51,59,60].

It is worth mentioning that the doses of probiotics were different between the included studies, the minimum dose being 10^6^ and the maximum dose 5 × 10^10^ CFU. One of the main reasons for this variations in the doses may be the characteristics of each probiotic. Some strains are more resistant to storage, and the dosage can also vary depending on how it is administered, for example either in dairy products or via capsule [61].

Another important point is the duration of the studies, which ranged from 4 to 36 weeks. However, most of the studies showing positive effects presented an intervention duration of 12 weeks. Therefore, 12 weeks seems to be the trend to start observing positive effects on body weight and/or fat mass by using probiotics. It should be noted that a reduction of ≥5% in initial body weight in a period of 6 months is clinically relevant and this would be associated with a significant reduction in some cardiovascular risk factors such as a reduction in blood pressure, lipids and blood glucose [62]. In the trial of Michael et al. [48], 40% of the participants in the probiotic group achieved this reduction after 9 months of supplementation, without dietary limitations.

Probiotics and synbiotics have been proposed to exert a decrease in body weight through different mechanisms (reviewed by [63,64]). Probiotics help in the recovery of the tight junctions between epithelial cells, thus reducing intestinal permeability, preventing the translocation of bacteria and reducing inflammation derived from lipopolysaccharides (LPS). The reduction in inflammation leads to an increase in insulin sensitivity in the hypothalamus, which improves satiety. Additionally, increased concentrations of leptin in adipose tissue, glucagon-like peptide 1 (GLP-1) and pancreatic polypeptide (PPY) in the intestine leads to a reduction in food intake due to an increase in satiety [15]. Recently, oral microbiota has also been associated to the development of obesity due to its modulating effects on the intestinal microbiota [65,66] and could have had an effect in the some of the observed results, especially in those where the intervention implied the ingestion of fermented foods.

Despite the observed beneficial effects, probiotics and synbiotics are not yet considered an alternative strategy in the treatment of obesity, probably due to the lack of regulation of this market [67]. In fact, in these products essential details such as the type of strain—or its combinations when more than one used—, the number of microorganisms included, the treatment duration, the route of administration, the formulation or the shelf-life and storage conditions, are often missing. Consequently, medical providers and the public are faced with a plethora of probiotic products with not proved health claims. In fact, the European Food Safety Authority (EFSA) has rejected all submitted health claims for probiotics so far. More evidence-based trials that support their use are needed, taking into account that even when evidence exists, not all probiotic products are equally effective for all disease prevention or treatment indications.

The present study had some limitations. First of all, in many of the studies the probiotic/synbiotic intervention was accompanied by dietary or physical activity interventions, which may have hidden the real effect of the probiotic strain(s) used. In addition, there were also variations in the populations included in the different studies regarding sex and age, which can introduce bias. The strengths of this study are that only randomized clinical trials were included in order to compilate the highest degree of evidence, conducted in otherwise healthy people with overweight or obesity in order to minimize biases. Additionally, the review includes recent studies and provides specific strains, doses and intervention times.

## 5. Conclusions

From the analyzed randomized clinical trials, this systematic review indicates that both probiotics and synbiotics, specifically certain strains of *Lactobacillus gasseri*, *L. rhamnosus*, *L. plantarum*, *L. curvatus* associated with other *Lactobacillus* species and/or with species from the *Bifidobacterium* genus, have the potential to aid in weight and fat mass loss in overweight and obese populations. There is still a need, though, for clinical trials, in order to state more accurate recommendations in terms of strains, doses and intervention times. It is also suggested to carry out studies in homogeneous populations in terms of sex and age. In addition to this, it would be ideal that future trials would be carried out in the absence of weight loss techniques (such as dietary recommendations for weight loss and physical activity programs), in order to evaluate the specific effect of the strain/s.

## Figures and Tables

**Table 1:** Detailed summary of the selected studies included in the revision.

Author	Year, Country	Population	Design	Intervention	Control	Strains & Doses	Inter Period	Results
Kadooka et al. [23]	2010, Japan	*n* = 87 *n*(IG) = 43 *n*(CG) = 44 Adults (male and female) Visceral fat 81.2–178.5 cm^2^ BMI: 24.2–30.7 kg/m^2^ Age: 33–63 years	Multi-center RCT-DB	200 g/day of fermented milk	200 g/day of fermented milk without *Lactobacillus gasseri* SBT2055	*Lactobacillus gasseri* SBT2055 (5 × 10^10^ CFU/100 g)	12 weeks	Body weight, BMI, waist and hip circumferences decreased significantly (*p* ≤ 0.001). In the active group the visceral and abdominal subcutaneous fat areas decreased significantly (*p* ≤ 0.01)
Omar et al. [24]	2013, Canada	*n* = 28 *n*(IG) = 14 *n*(GC) = 14 Adults (male and female) BMI: 25–32 kg/m^2^ Age: 18–60 years	Cross-over RCT-DB	(i) Yogurt with probiotic 1 (ii) Yogurt with probiotic 2 Both groups were put on diet	Control yogurt	(i) 100 g of control yogurt + 10 g of 1.39 × 10^9^ CFU microencapsulated BSH-active *Lactobacillus acidophilus* (ii) 100 g of control yogurt + 10 g of 1.08 × 10^9^ CFU microencapsulated FAE-active *Lactobacillus fermentum*	13 weeks	No significant differences in body weight were observed at baseline or endpoint across the three treatments. Significant reductions in total fat mass by 3% from baseline (*p* = 0.05)
Sanchez et al. [25]	2013, Canada	*n* = 125 *n*(IG) = 62 *n*(GC) = 63 Healthy adults (male and female) BMI: 29–41 kg/m^2^ Age:18–55 years	RCT-DB	2 capsules/day of probiotic + moderate restriction of energy in the first 12 weeks followed by 12 weeks of maintenance	Placebo	*Lactobacillus rhamnosus* CGMCC1.3724 (LPR) (1.62 × 10^8^ CFU) with 300 mg of one mix of oligofructose and inulin (70:30, vv)	24 weeks	No significant reduction in weight among the comparison groups. Significant interaction between sex and intervention. Weight loss in female in the intervention group was significantly higher than those of placebo group (*p* = 0.02)
Safavi et al. [26]	2013, Iran	*n* = 70 *n*(IG) = 29 *n*(CG) = 27 Healthy children and adolescents BMI: ≥85th percentile	RCT-TB	1 capsule/day of the synbiotic	Placebo of maltodextrin	*Lactobacillus casei*, *Lactobacillus rhamnosus*, *Streptococcus thermophilus*, *Bifidobacterium breve*, *Lactobacillus acidophilus*, *Bifidobacterium longum* and *Lactobacillus bulgaricus* of human origin with prebiotics (fructo-oligosaccharides), vitamins A, C and E. Every capsule contained 2 × 10^8^ CFU of probiotic bacteria	8 weeks	The decrease in the z scores of the BMI (*p* = 0.002), waist circumference (*p* ≤ 0.0001) and the waist-to-hip ratio (*p* ≤ 0.0001) were significantly higher in the synbiotic group than in the placebo group
Kadooka et al. [27]	2013, Japan	*n* = 210 *n*(IG) = 140 (69/71) *n*(CG) = 70 Healthy adults (male and female) Average BMI 27 kg/m^2^ and with large areas of visceral fat (80.2–187.8 cm^2^) Age: 25–60 years	Multi-center RCT-DB	200 g/day of milk fermented with strains of probiotics to different levels. Participants maintained their life style, including diet and exercise	200 g fermented milk without probiotic	Starter cultures: lactic acid bacteria (*Streptococcus thermophilus* and *Lactobacillus delbrueckii* ssp.) and cells of *Lactobacillus gasseri* SBT2055 (LG2055) to levels of 10^6^, 10^7^ (CFU/g)	12 weeks	Significant decrease in the areas of visceral fat, BMI, waist and hip circumference (*p* ≤ 0.01) in the groups with doses of 10^7^ and 10^6^ (CFU/g) at weeks 8 and 12
Zarrati et al. [28]	2014, Iran	*n* = 75 *n*(IG) = 25/25 *n*(GC) = 25 Healthy adults with overweight or obesity BMI > 25 kg/m^2^ Age: 20–50 years	RCT-DB	(i) Low calorie diet with probiotic yogurt (200 g/day) (ii) Consumption of the same probiotic yogurt (200 g/day) without the diet low in calories	Low calorie diet with regular yogurt consumption (200 g/day)	*Lactobacillus acidophilus* La5, *Bifidobacterium* BB12 and *Lactobacillus casei* DN001 10^8^ CFU/g	8 weeks	A reduction in the BMI, percentage of fat and leptin level, which was more evident in the groups that received the weight loss diet with probiotic yogurt. Significant differences in weight, BMI and waist circumference between groups (*p* = 0.001)
Lee et al. [29]	2014, Korea Republic	*n* = 50 *n*(IG) = 25 *n*(GC) = 25 Healthy female Waist circumference > 85 cm, BMI > 25 kg/m^2^ Age: 19–65 years	RCT-DB	Supplementation 2 times/day with 3 g of *Bofutsushosan* (BTS) and DUOLAC 7 probiotic. It was suggested to the participants to limit energy intake to 20–25 kcal/kg	3 g of *Bofutsushosan* (BTS) and placebo capsules 2 times/day	DUOLAC 7: 5000 million viable cells of *Streptococcus thermophilus* (KCTC 11870BP), *Lactobacillus plantarum* (KCTC 10782BP), *Lactobacillus acidophilus* (KCTC 11906BP), *Lactobacillus rhamnosus* (KCTC 12202BP), *Bifidobacterium lactis* (KCTC 11904BP), *Bifidobacterium longum* (KCTC 12200BP) and *Bifidobacterium breve* (KCTC 12201BP)	8 weeks	Both groups showed significant reductions in weight and waist circumference (*p* = 0.000). No significant differences were observed in body composition
Jung et al. [30]	2015, Korea Republic	*n* = 120 *n*(IG) = 60 *n*(CG) = 60 Adults (male and female) BMI: 25–30 kg/m^2^ Age 20–65 years	RCT-DB	2 g probiotic powder, two times/day (immediately after breakfast and dinner). Participants maintained their diet and normal lifestyle	2 g of powder containing 1.34 g of crystalline cellulose, 0.6 g of lactose and 0.06 g of blueberry	2 g powder of strains *Lactobacillus curvatus* HY7601 and *Lactobacillus plantarum* KY1032, each at 2.5 × 10^9^ CFU/capsule	12 weeks	The probiotic group showed reductions in body weight (*p* = 0.008), BMI (*p* = 0.006) and waist circumference (*p* = 0.015) in relation to the initial value. When the changes were compared anthropometrically (differences with relative to baseline) between control and probiotics groups, the group of probiotics had greater reductions in body weight (*p* = 0.001) and BMI (*p* = 0.001)
Ipar et al. [31]	2015, Turkey	*n =* 86 *n*(IG) = 43 *n*(CG) = 43 Children with primary obesity Age: 4–17 years	Open label RCT	Supplementation with synbiotic and 10% caloric reduction and increase in physical activity	10% caloric reduction and increase in physical activity	Probiotics: *Lactobacillus acidophilus* (4.3 × 10^8^ CFU/sachet), *Lactobacillus rhamnosus* (4.3 × 10^8^ CFU/sachet), *Bifidobacterium bifidum* (4.3 × 10^8^ CFU/sachet), *Bifidobacterium longum* (4.3 × 10^8^ CFU/sachet), *Enterococcus faecium* (8.2 × 10^8^ CFU/sachet). Prebiotics: fructo-oligosaccharides (FOS) 625 mg, lactulose 400 mg	4 weeks	One month of supplementation with synbiotic resulted in significant weight reduction (*p* ≤ 0.001) and BMI (*p* ≤ 0.01)
Stenman et al. [32]	2016, Finland	*n =* 225 *n*(IG) = 168 (57/55/56) *n*(CG) = 57 Adults (male and female) Waist hip ratio *≥* 0.88 for female, BMI: 28–34.9 kg/m^2^ Age: 18–65 years	RCT-DB	(i). Probiotic *Bifidobacterium animalis *ssp*. lactis* 420 (B420) (ii). Probiotic of polydextrose (LU) (iii). Probiotic *Bifidobacterium animalis *ssp*. lactis* 420 (B420) + Probiotic (LU + B420)	Placebo, cellulose microcrystalline 12 g/day	B420, 10^10^ CFU/day prebiotic of polydextrose 12 g/day B420, 10^10^ CFU/day in 12 g of polydextrose	24 weeks	The probiotic B420 and the synbiotic LU + B420 seemed to improve weight control in the population analyzed by protocol due to changes in body fat mass (*p* = 0.02)
Madjd et al. [33]	2016, Iran	*n =* 89 *n*(IG) = 44 *n*(CG) = 45 Premenopausal obese or overweight female, healthy BMI: 27–40 kg/m^2^ Age: 18–50 years	RCT-SB	400 g/day of yogurt probiotic, enriched with culture. Both groups are put on a diet for weight loss and physical activity	400 g/day standard yogurt low in fat with main meals	Starter cultures: *Streptococcus thermophilus* and *Lactobacillus Bulgaricus.* Probiotic: *Lactobacillus acidophilus* LA5 and *Bifidobacterium lactis* BB12, with a minimum total of 1 × 10^7^ CFU.	12 weeks	No significant differences were observed in weight loss and anthropometric measurements between groups after the intervention
Higashikawa et al. [34]	2016, Japan	*n =* 62 *n*(IG) = 21/21 *n*(GC) = 20 Adults (male and female), healthy BMI: 25–30 kg/m^2^ Age: 20–70 years	RCT-DB	Powder of *Pediococcus pentosaceus* LP28 alive with dextrin. Powder of heat-killed *Pediococcus pentosaceus* LP28 with dextrin.	Placebo (dextrin)	Live LP29 7.5 mL 10^10^ CFU Dead LP29 7.5 mL 10^10^ CFU	12 weeks	The LP28 removed by heat showed significant reductions in the BMI (*p* = 0.035), body fat percentage (*p* = 0.002), body fat mass (*p* = 0.004) and waist circumference (*p* = 0.009).
Gomes et al. [35]	2017, Brazil	*n =* 43 *n*(IG) = 21 *n*(GC) = 22 Female with overweight or obesity, healthy BMI: 24.9–40 kg/m^2^ Age: 20–59 years	RCT-DB	4 sachet of probiotic daily before breakfast+ dietary intervention	Placebo+ Dietary intervention	1 × 10^9^ CFU of each of the probiotic strains: *Lactobacillus acidophilus* LA-14, *Lactobacillus casei* LC-11, *Lactococcus lactis* LL-23, *Bifidobacterium bifidum* BB-06, *Bifidobacterium lactis* BL-4 (Danisco). Totaling 2 × 10^10^ CFU/day	8 weeks	Dietary intervention+ probiotic group showed greater reductions in waist circumference (*p* = 0.03), waist-height ratio (*p* = 0.02), conicity index (*p* = 0.03) in comparison with the dietary intervention
Mohammadi-Sartang et al. [36]	2018, Iran	*n =* 94 *n*(IG) = 44 *n*(CG) = 43 Adults (male and female) BMI: 25–34.9 kg/m^2^ Age: 20–65 years	RCT-DB	Two daily servings (2 × 250 g) of fortified yogurt containing 5 g protein powder, 3 g inulin as a prebiotic, 500 mg calcium and 500 IU vitamin D3. All participants received a diet energy restriction for the study intervention 500 kcal less, with a composition of macronutrients of 55% of carbohydrates, 15% of protein and 30% fat	Two daily servings (2 × 250 g) of low fat natural yogurt, that contained 300 mg of calcium. Starter cultures: *Streptococcus thermophilus* and *Lactobacillus bulgaricus*	Starter cultures: *S. thermophilus* and *L. Bulgaricus* enriched with at least 10^7^ CFU/g of *Bifidobacterium lactis* Bb-12	10 weeks	Decreases in BMI (kg/m^2^), waist circumference (cm), body fat mass (kg) and body fat percentage (%) in both groups at the end of the study comparison with the initial values. Reductions in waist circumference (*p* = 0.002), body fat (*p* = 0.023) and body fat percentage (*p* = 0.028) higher in the fortified yogurt group compared to low-fat yogurt group
Kianifar et al. [37]	2018, Iran	*n =* 46 *n*(IG) = 23 *n*(CG) = 23 Kids (male and female) with obesity BMI: ≥85th percentile Age: 7–13 years	Pilot-Study RCT-DB	Restrictive diet, physical activity plan and 1 capsule of synbiotics per day	Same as the treatment group, received a restrictive diet and physical activity plan but with a capsule of placebo per day	Fructo-oligosaccharide, vitamins A, C and E, 10^8^ UFC of a combination of *Lactobacillus casei*, *Lactobacillus rhamnosus*, *Streptococcus thermophilus*, *Bifidobacterium breve*, *Lactobacillus acidophilus*, *Bifidobacterium infantis* and *Lactobacillus bulgaricus*	12 weeks	Significant reductions in z score of BMI and percentage of fat in both groups (*p* ≤ 0.001). Waist circumference decreased significantly only in the group intervened with synbiotics (*p* ≤ 0.001).
Kim et al. [38]	2018, Korea Republic	*n =* 90 *n*(IG) = 60 (30/30) *n*(CG) = 30 Adults without comorbidities with overweight or obesity BMI: 25–35 kg/m^2^ Age: 20–75 years	RCT-DB	Two capsules/day (400 mg/capsule) low dose or high of probiotic. Both groups reduced 200 kcal/day their energy intake and increased by 100 kcal/day their physical activity, during intervention period	Two capsules (400 mg/capsule) of placebo that composed of maltodextrin, crystalline cellulose and magnesium stearate	*Lactobacillus gasseri*, low-dose BNR17 (10^9^ CFU) or high dose (10^10^ CFU	12 weeks	BMI, hip circumference and waist–hip ratio were not significantly different between groups at weeks 0, 6, and 12. The waist circumference in the intervened groups and hip circumference in the low dose group decreased significantly after BNR17 consumption for 12 weeks within each group (*p* = 0.045, 0.012 and 0.033, respectively).
Pedret et al. [39]	2018, Spain	*n =* 126 *n*(IG) = 86 (42/44) *n*(CG) = 40 Adults (male and female) Abdominal obesity (circumference of waist *≥* 102 cm for female) Age > 18 years	RCT-DB	1 capsule per day of the following probiotics: *Bifidobacterium animalis* CECT 8145, heat killed *Bifidobacterium animalis* 8145. Dietary recommendations were made according to the 2013 guidelines of Adults Treatment Panel (ATP III).	Placebo (300 mg of maltodextrin)	(i) 100 mg of the live strain, 10^10^ CFU/capsule containing 200 mg of maltodextrin (ii) 100 mg/capsule of CECT strain 8145 killed by heat to a concentration of 10^10^ CFU before thermal treatment, which contained 200 mg of maltodextrin	12 weeks	Treatment with Ba8145 decreased the BMI as compared to its initial value and the placebo group (*p* ≤ 0.05). Both interventions by Ba8145 decreased the waist circumference, the ratio of waist circumference/height and the conicity index (*p* ≤ 0.05), relative to its initial value. The changes relative to the placebo group were also significant (*p* ≤ 0.05)
Sudha et al. [40]	2019, India	*n =* 90 *n*(IG) = 45 *n*(CG) = 45 Adults (male and female), healthy BMI: 25–32 kg/m^2^ Age: 30–65 years	RCT-DB	Two capsules/day of probiotic UB0316	Placebo of maltodextrin.	UB0316: *Lactobacillus salivarius* UBLS-22, *Lactobacillus casei* UBLC42 *Lactobacillus plantarum*, UBLP-40 *Lactobacillus acidophilus* UBLA-34 *Bifidobacterium breve UBBr-01*, *Bacillus coagulans* Unique IS2, 5 10^9^ CFU/capsule, and 100 mg of fructo- oligosaccharides	12 weeks	At 12 weeks, supplementation of UB0316 showed significant reductions in BMI (*p* = 0.0001), body weight (*p* ≤ 0.0001), and in the waist-to-hip ratio (*p* = 0.007), compared to the placebo group
Hadi et al. [41]	2019, Iran	*n =* 60 *n*(IG) = 30 *n*(CG) = 30 Adults (male and female) BMI: 25–35 kg/m^2^ Age: 20–50 years	RCT-DB	Consumption of a synbiotic capsule per day of 500 mg. Participants maintained their diet and normal lifestyle	Placebo (starch)	*Lactobacillus acidophilus*, *Lactobacillus casei*, *Bifidobacterium bifidum* (2 × 10^9^ CFU/capsule), inulin	8 weeks	Significant decrease in body weight (*p* = 0.03). Trend towards a significant decrease in BMI (*p* = 0.06) and waist circumference (*p* = 0.08) compared to the control group
Gutiérrez-Repiso et al. [42]	2019, Spain	*n =* 33 *n*(IG) = 24 (15/9) *n*(GC) = 9 Adults (male and female) BMI *≥* 30 kg/m^2^	RCT-SB	All participants underwent a weight loss program of two phases. Phase 1: ketogenic diet very low in calories (VLCKD) with supplementation of vitamins and minerals + synbiotic. Phase 2: low calorie diet (LCD) + synbiotic 2	Phase 1: placebo Phase 2: the control group split in two: one continued receiving the placebo (control) and the other group received synbiotic 2 (placebo group + synbiotic 2)	Synbiotic phase 1: *Bifidobacterium lactis*, *Lactobacillus rhamnosus*, *Bifidobacterium longum* ES1 and prebiotic fiber. Synbiotic phase 2: *Bifidobacterium animalis *subsp*. lactis* and prebiotic fiber	16 weeks (8 weeks each phase)	In all three treatments, the caloric restriction induced significant changes in weight, waist circumference and BMI during the entire intervention. In the group placebo-synbiotic 2, the weight loss percentage was significantly higher than in the group of synbiotic 1-synbiotic 2 (*p* = 0.030)
Michael et al. [43]	2020, Bulgaria	*n =* 220 *n*(IG) = 110 *n*(GC) = 110 Healthy adults (male and female) Waist circumference > 100 cm in male and >89 cm in female. BMI: 25–39.4 kg/m^2^ Age: 30–65 years	RCT-DB	Consumption of a capsule of the probiotic Lab4P. Participants kept their usual life style	Placebo of cellulose microcrystalline	Lab4P: *Lactobacillus acidophilus* CUL60 (NCIMB 30157), *Lactobacillus acidophilus* CUL21 (NCIMB 30156), *Lactobacillus plantarum* CUL66 (NCIMB 30280), *Bifidobacterium bifidum* CUL20 (NCIMB 30153) and *Bifidobacterium animalis *subsp*. Lactis* CUL34 (NCIMB 30172) for a total of 5 × 10^10^ (CFU) per capsule	24 weeks	Significant decrease in weight between groups (*p* ≤ 0.0001), BMI (*p* ≤ 0.0001), waist circumference (*p* ≤ 0.0001) and the ratio waist/height (*p* ≤ 0.0001)
Razmpoosh et al. [44]	2020, Iran	*n =* 70 *n*(IG) = 35 *n*(CG) = 35 Women with overweight and obesity, non-smokers. BMI *≥* 25 kg/m^2^	RCT	Participants received a diet low in energy with 50 g/day of kashk yogurt (high in protein, calcium and enriched with probiotics)	Diet low in energy without kashk	1.85 × 10^6^ (CFU/g) *L. acidophilus* La5 and 1.79 × 10^6^ CFU/g of *B*. *lactis* Bb12	8 weeks	Significant reductions in the intervention group in BMI (*p* = 0.018), percentage of body fat (*p* = 0.037) and waist circumference (*p* = 0.047) in comparison with the control group
Song et al. [45]	2020, Korea Republic	*n =* 50 *n*(IG) = 25 *n*(GC) = 25 Adults (male and female) healthy, with obesity (agree with the Obesity guidelines of Asia-Pacific) BMI > 25 kg/m^2^ Age: 20–60 years	RCT-DB	2 probiotics capsules/day	Placebo of fructo-oligosaccharide and magnesium. stearate.	*Bifidobacterium breve* CBT BR3 isolated from Korean infant stools (15 billion viable cells/2 capsules), *Lactobacillus plantarum* CBT LP3 isolated from kimchi, Korean fermented vegetable products (15 billion viable cells/2 capsules)	12 weeks	Significantly reduction of waist circumference (*p* = 0.049) and the relationship between visceral and subcutaneous fat area (*p* ≤ 0.001) in the probiotics group
Lim et al. [46]	2020, Korea Republic	*n =* 114 *n*(IG) = 57 *n*(CG) = 57 Adults (male and female), healthy BMI > 25 kg/m^2^ Age: 20–65 years	RCT-DB	2 probiotic capsules per day. Healthy life style recommendations were made and the participants were encouraged to maintain a favorable lifestyle	Placebo	*Lactobacillus sakei* CJLS03 5 × 10^9^ CFU	12 weeks	Body fat mass decreased by 0.2 kg in the probiotic group and increased by 0.6 kg in the placebo group (*p* = 0.018). After 12 weeks, the waist circumference was 0.8 cm smaller in the CJLS03 group than in the placebo group (*p* = 0.013). BMI and body weight did not change after 12 weeks
Sergeev et al. [47]	2020, USA	*n =* 20 *n*(IG) = 10 *n*(GC) = 10 Adults (male and female) with overweight or obesity Average BMI: 33.5 kg/m^2^ Age: 47.4 years	RCT	A weight-loss eating plan was followed (low in carbohydrates high in protein), plus a capsule of synbiotic per day	The same eating plan as the intervention group was followed placebo group, but received a placebo capsule per day	One capsule contained: 15 × 10^9^ CFU of patented strains of *Lactobacillus acidophilus* DDS-1, *Bifidobacterium lactis* UABla-12, *Bifidobacterium longum* UABl-14 and *Bifidobacterium bifidum* UABb-10. The prebiotic component was a mix of trans-galacto-oligosaccharides (GOS) at a dose of 5.5 g/day	12 weeks	No statistically significant differences in the body composition (body mass, BMI, body fat mass, percentage of body fat, lean body mass) between placebo and synbiotic groups at the end of clinical trial
Michael et al. [48]	2021, Bulgaria	*n =* 70 *n*(IG) = 35 *n*(CG) = 35 Adults (male and female) with overweight, healthy BMI: 25–29.9 kg/m^2^ Waist circumference of males> 100 cm; females > 89 cm Age: 45–65 years	RCT-DB	Daily consumption of a probiotic Lab4P capsule. Participants maintained their diet and normal lifestyle	Placebo	Lab4P: *Lactobacillus acidophilus* CUL60 (NCIMB 30157, *Lactobacillus acidophilus* CUL21 (NCIMB 30156), *Lactobacillus plantarum* CUL66 (NCIMB 30280), *Bifidobacterium bifidum* CUL2, *Bifidobacterium animalis *subsp*. Lactis* CUL34 (NCIMB 30172) for a total of 5 × 10^10^ CFU/capsule	36 weeks	Significant decrease in body weight (*p* ≤ 0.0001) between groups, predominantly in the probiotic group. Significant decrease among groups in waist and hip circumference (*p* < 0.0001)
Rahayu et al. [49]	2021, Indonesia	*n =* 60 *n*(IG) = 30 *n*(CG) = 30 Adults (male and female) healthy BMI *≥* 25 kg/m^2^	RCT	1 g of powdered skimmed milk with probiotic	1 g of powdered skimmed milk without probiotic	*Lactobacillus plantarum* Dad-13 of 2 × 10^9^ CFU/pack	12 weeks	Significant decrease in body weight and BMI (*p* ≤ 0.05) after 90 days of ingestion of probiotics

*n*(IG): number of participants in the intervention group; *n*(GC): number of participants in the control group; RCT: Randomized Controlled Trial; SB: Simple Blind; DB: Double Blind; TB: Triple Blind; BMI: Body Mass Index.

## Data Availability

The publications analyzed for this systematic study can be accessed from their respective journals, whereby access restrictions may apply.

## Supplementary Materials

The following are available online at https://www.mdpi.com/article/10.3390/nu13103627/s1, Table S1: Search strategies used for this systematic review, Table S2: Assessment of the methodological quality of the included clinical trials, using the Jadad scale.

## Abbreviations

The following abbreviations are used in this manuscript:

BMI	Body Mass Index
CFU	Colony Forming Unit
Fiaf	Fasting-induced adipose factor
GLP	Glucagon-like peptide
IM	Intestinal Microbiota
ISAPP	International Scientific Association of Probiotics and Prebiotics
LAB	Lactic Acid Bacteria
LPS	Lipopolysaccharides
PPY	Pancreatic polypeptide
PCOS	Polycystic Ovary Syndrome
RCT	Randomized Controlled Trial
RCT-DB	Randomized Controlled Trial Double Blind
RCT-TB	Randomized Controlled Trial Triple Blind
WHO	World Health Organization

## References

[1] WHO Obesity and Overweight. https://www.who.int/news-room/fact-sheets/detail/obesity-and-overweight.

[2] Roth G.A., Abate D., Abate K.H., Abay S.M., Abbafati C., Abbasi N., Abbastabar H., Abd-Allah F., Abdela J., Abdelalim A. (2018). Global, regional, and national age-sex-specific mortality for 282 causes of death in 195 countries and territories, 1980–2017: A systematic analysis for the Global Burden of Disease Study 2017. Lancet.

[3] Wright S.M., Aronne L.J. (2012). Causes of obesity. Abdom. Radiol..

[4] Blüher M. (2019). Obesity: Global epidemiology and pathogenesis. Nat. Rev. Endocrinol..

[5] María Magdalena Farías N., Catalina Silva B., Jaime Rozowski N. (2011). Microbiota Intestinal: Rol en Obesidad. Rev. Chil. Nutr..

[6] Muscogiuri G., Cantone E., Cassarano S., Tuccinardi D., Barrea L., Savastano S., Colao A. (2019). Gut microbiota: A new path to treat obesity. Int. J. Obes. Suppl..

[7] Angelakis E., Armougom F., Million M., Raoult D. (2012). The relationship between gut microbiota and weight gain in humans. Future Microbiol..

[8] Cani P.D., Delzenne N.M. (2009). The Role of the Gut Microbiota in Energy Metabolism and Metabolic Disease. Curr. Pharm. Des..

[9] Le Chatelier E., Nielsen T., Qin J., Prifti E., Hildebrand F., Falony G., Almeida M., Arumugam M., Batto J.-M., Kennedy S. (2013). Richness of human gut microbiome correlates with metabolic markers. Nature.

[10] Million M., Maraninchi M., Henry M., Armougom F., Richet H., Carrieri P., Valero R., Raccah D., Vialettes B., Raoult D. (2011). Obesity-associated gut microbiota is enriched in Lactobacillus reuteri and depleted in Bifidobacterium animalis and Methanobrevibacter smithii. Int. J. Obes..

[11] Turnbaugh P.J., Ley R.E., Mahowald M.A., Magrini V., Mardis E.R., Gordon J.I. (2006). An obesity-associated gut microbiome with increased capacity for energy harvest. Nat. Cell Biol..

[12] Ley R.E., Bäckhed F., Turnbaugh P., Lozupone C.A., Knight R.D., Gordon J.I. (2005). Obesity alters gut microbial ecology. Proc. Natl. Acad. Sci. USA.

[13] Parekh P.J., Arusi E., Vinik A.I., Johnson D.A. (2014). The Role and Influence of Gut Microbiota in Pathogenesis and Management of Obesity and Metabolic Syndrome. Front. Endocrinol..

[14] Baothman O.A., Zamzami M.A., Taher I., Abubaker J., Abu-Farha M. (2016). The role of Gut Microbiota in the development of obesity and Diabetes. Lipids Health Dis..

[15] Carvalho B.M., Saad M.J.A. (2013). Influence of Gut Microbiota on Subclinical Inflammation and Insulin Resistance. Mediat. Inflamm..

[16] Hill C., Guarner F., Reid G., Gibson G.R., Merenstein D.J., Pot B., Morelli L., Canani R.B., Flint H.J., Salminen S. (2014). Expert Consensus Document: The International Scientific Association for Probiotics and Prebiotics consensus statement on the scope and appropriate use of the term probiotic. Nat. Rev. Gastroenterol. Hepatol..

[17] Gibson G.R., Hutkins R., Sanders M.E., Prescott S.L., Reimer R.A., Salminen S.J., Scott K., Stanton C., Swanson K.S., Cani P.D. (2017). Expert consensus document: The International Scientific Association for Probiotics and Prebiotics (ISAPP) consensus statement on the definition and scope of prebiotics. Nat. Rev. Gastroenterol. Hepatol..

[18] Swanson K.S., Gibson G.R., Hutkins R., Reimer R.A., Reid G., Verbeke K., Scott K.P., Holscher H.D., Azad M.B., Delzenne N.M. (2020). The International Scientific Association for Probiotics and Prebiotics (ISAPP) consensus statement on the definition and scope of synbiotics. Nat. Rev. Gastroenterol. Hepatol..

[19] Liberati A., Altman D.G., Tetzlaff J., Mulrow C., Gøtzsche P.C., Ioannidis J.P.A., Clarke M., Devereaux P.J., Kleijnen J., Moher D. (2009). The PRISMA statement for reporting systematic reviews and meta-analyses of studies that evaluate health care interventions: Explanation and elaboration. J. Clin. Epidemiol..

[20] Yumuk V., Tsigos C., Fried M., Schindler K., Busetto L., Micic D., Toplak H. (2015). European Guidelines for Obesity Management in Adults. Obes. Facts.

[21] Dickey R.A., Bartuska C.G.D., Bray F.W.G., Callaway M.C.W., Davidson F.T.E., Feld M.S., Ferraro M.T.R., Hodgson S.F., Jellinger F.S.P., Kennedy F.P.F. (1998). AACE/ACE Position Statement on the Prevention, Diagnosis, and Treatment of Obesity (1998 Revision). Endocr Pract..

[22] Jadad A.R., Moore R.A., Carroll D., Jenkinson C., Reynolds D.J.M., Gavaghan D.J., McQuay H.J. (1996). Assessing the quality of reports of randomized clinical trials: Is blinding necessary?. Control. Clin. Trials.

[23] Kadooka Y., Sato M., Imaizumi K., Ogawa A., Ikuyama K., Akai Y., Okano M., Kagoshima M., Tsuchida T. (2010). Regulation of abdominal adiposity by probiotics (Lactobacillus gasseri SBT2055) in adults with obese tendencies in a randomized controlled trial. Eur. J. Clin. Nutr..

[24] Omar J.M., Chan Y.-M., Jones M.L., Prakash S., Jones P.J.H. (2013). Lactobacillus fermentum and Lactobacillus amylovorus as probiotics alter body adiposity and gut microflora in healthy persons. J. Funct. Foods.

[25] Sanchez M., Darimont C., Drapeau V., Emady-Azar S., Lepage M., Rezzonico E., Ngom-Bru C., Berger B., Philippe L., Ammon-Zuffrey C. (2014). Effect of Lactobacillus rhamnosusCGMCC1.3724 supplementation on weight loss and maintenance in obese men and women. Br. J. Nutr..

[26] Safavi M., Farajian S., Kelishadi R., Mirlohi M., Hashemipour M. (2013). The effects of synbiotic supplementation on some cardio-metabolic risk factors in overweight and obese children: A randomized triple-masked controlled trial. Int. J. Food Sci. Nutr..

[27] Kadooka Y., Sato M., Ogawa A., Miyoshi M., Uenishi H., Ogawa H., Ikuyama K., Kagoshima M., Tsuchida T. (2013). Effect of Lactobacillus gasseri SBT2055 in fermented milk on abdominal adiposity in adults in a randomised controlled trial. Br. J. Nutr..

[28] Zarrati M., Salehi E., Nourijelyani K., Mofid V., Zadeh M.J.H., Najafi F., Ghaflati Z., Bidad K., Chamari M., Karimi M. (2014). Effects of Probiotic Yogurt on Fat Distribution and Gene Expression of Proinflammatory Factors in Peripheral Blood Singlenuclear Cells in Overweight and Obese People with or without Weight-Loss Diet. J. Am. Coll. Nutr..

[29] Lee S.J., Bose S., Seo J.-G., Chung W.-S., Lim C.-Y., Kim H. (2014). The effects of co-administration of probiotics with herbal medicine on obesity, metabolic endotoxemia and dysbiosis: A randomized double-blind controlled clinical trial. Clin. Nutr..

[30] Jung S., Lee Y.J., Kim M., Kim M., Kwak J.H., Lee J.W., Ahn Y.-T., Sim J.-H., Lee J.H. (2015). Supplementation with two probiotic strains, Lactobacillus curvatus HY7601 and Lactobacillus plantarum KY1032, reduced body adiposity and Lp-PLA2 activity in overweight subjects. J. Funct. Foods.

[31] Ipar N., Aydogdu S.D., Yildirim G.K., Inal M., Gies I., Vandenplas Y., Dinleyici E.C. (2015). Effects of synbiotic on anthropometry, lipid profile and oxidative stress in obese children. Benef. Microbes.

[32] Hibberd A.A., Yde C.C., Ziegler M.L., Honoré A.H., Saarinen M.T., Lahtinen S., Stahl B., Jensen H.M., Stenman L.K. (2019). Probiotic or synbiotic alters the gut microbiota and metabolism in a randomised controlled trial of weight management in overweight adults. Benef. Microbes.

[33] Madjd A., Taylor M.A., Neek L.S., Delavari A., Malekzadeh R., Macdonald I.A., Farshchi H.R. (2016). Effect of weekly physical activity frequency on weight loss in healthy overweight and obese women attending a weight loss program: A randomized controlled trial. Am. J. Clin. Nutr..

[34] Higashikawa F., Noda M., Awaya T., Danshiitsoodol N., Matoba Y., Kumagai T., Sugiyama M. (2016). Antiobesity effect of Pediococcus pentosaceus LP28 on overweight subjects: A randomized, double-blind, placebo-controlled clinical trial. Eur. J. Clin. Nutr..

[35] Gomes A.C., De Sousa R.G.M., Botelho P.B., Gomes T.L.N., Prada P.D.O., Mota J.F. (2017). The additional effects of a probiotic mix on abdominal adiposity and antioxidant Status: A double-blind, randomized trial. Obesity.

[36] Mohammadi-Sartang M., Bellissimo N., de Zepetnek J.O.T., Brett N.R., Mazloomi S.M., Fararouie M., Bedeltavana A., Famouri M., Mazloom Z. (2018). The effect of daily fortified yogurt consumption on weight loss in adults with metabolic syndrome: A 10-week randomized controlled trial. Nutr. Metab. Cardiovasc. Dis..

[37] Kianifar H.R., Ahanchian H., Safarian M., Javid A., Farsad-Naeimi A., Jafari A., Kiani M.A., Dahri M. (2018). Effects of Synbiotics on Anthropometric Indices of Obesity in Children: A randomized double-blind placebo-controlled pilot study. Top. Clin. Nutr..

[38] Kim J., Yun J.M., Kim M.K., Kwon O., Cho B. (2018). Lactobacillus gasseri BNR17 Supplementation Reduces the Visceral Fat Accumulation and Waist Circumference in Obese Adults: A Randomized, Double-Blind, Placebo-Controlled Trial. J. Med. Food.

[39] Pedret A., Valls R.M., Calderón-Pérez L., Llauradó E., Companys J., Pla-Pagà L., Moragas A., Martín-Luján F., Ortega Y., Giralt M. (2019). Effects of daily consumption of the probiotic Bifidobacterium animalis subsp. lactis CECT 8145 on anthropometric adiposity biomarkers in abdominally obese subjects: A randomized controlled trial. Int. J. Obes..

[40] Sudha M.R., Ahire J.J., Jayanthi N., Tripathi A., Nanal S. (2019). Effect of multi-strain probiotic (UB0316) in weight management in overweight/obese adults: A 12-week double blind, randomised, placebo-controlled study. Benef. Microbes.

[41] Hadi A., Sepandi M., Marx W., Moradi S., Parastouei K. (2019). Clinical and psychological responses to synbiotic supplementation in obese or overweight adults: A randomized clinical trial. Complement. Ther. Med..

[42] Gutiérrez-Repiso C., Hernández-García C., García-Almeida J.M., Bellido D., Martín-Núñez G.M., Sánchez-Alcoholado L., Alcaide-Torres J., Sajoux I., Tinahones F.J., Moreno-Indias I. (2019). Effect of Synbiotic Supplementation in a Very-Low-Calorie Ketogenic Diet on Weight Loss Achievement and Gut Microbiota: A Randomized Controlled Pilot Study. Mol. Nutr. Food Res..

[43] Michael D.R., Jack A.A., Masetti G., Davies T.S., Loxley K.E., Kerry-Smith J., Plummer J.F., Marchesi J.R., Mullish B.H., McDonald J. (2020). A randomised controlled study shows supplementation of overweight and obese adults with lactobacilli and bifidobacteria reduces bodyweight and improves well-being. Sci. Rep..

[44] Razmpoosh E., Zare S., Fallahzadeh H., Safi S., Nadjarzadeh A. (2020). Effect of a low energy diet, containing a high protein, probiotic condensed yogurt, on biochemical and anthropometric measurements among women with overweight/obesity: A randomised controlled trial. Clin. Nutr. ESPEN.

[45] Song E.-J., Han K., Lim T.-J., Lim S., Chung M.-J., Nam M.H., Kim H., Nam Y.-D. (2020). Effect of probiotics on obesity-related markers per enterotype: A double-blind, placebo-controlled, randomized clinical trial. EPMA J..

[46] Lim S., Moon J.H., Shin C.M., Jeong D., Kim B. (2020). Effect of Lactobacillus sakei, a Probiotic Derived from Kimchi, on Body Fat in Koreans with Obesity: A Randomized Controlled Study. Endocrinol. Metab..

[47] Sergeev I.N., Aljutaily T., Walton G., Huarte E. (2020). Effects of Synbiotic Supplement on Human Gut Microbiota, Body Composition and Weight Loss in Obesity. Nutrients.

[48] Michael D.R., Davies T.S., Jack A.A., Masetti G., Marchesi J.R., Wang D., Mullish B.H., Plummer S.F. (2021). Daily supplementation with the Lab4P probiotic consortium induces significant weight loss in overweight adults. Sci. Rep..

[49] Rahayu E.S., Mariyatun M., Manurung N.E.P., Hasan P.N., Therdtatha P., Mishima R., Komalasari H., Mahfuzah N.A., Pamungkaningtyas F.H., Yoga W.K. (2021). Effect of probiotic Lactobacillus plantarum Dad-13 powder consumption on the gut microbiota and intestinal health of overweight adults. World J. Gastroenterol..

[50] Madjd A., Taylor M.A., Mousavi N., Delavari A., Malekzadeh R., Macdonald I.A., Farshchi H.R. (2016). Comparison of the effect of daily consumption of probiotic compared with low-fat conventional yogurt on weight loss in healthy obese women following an energy-restricted diet: A randomized controlled trial1. Am. J. Clin. Nutr..

[51] Stenman L.K., Lehtinen M.J., Meland N., Christensen J.E., Yeung N., Saarinen M.T., Courtney M., Burcelin R., Lähdeaho M.-L., Linros J. (2016). Probiotic With or Without Fiber Controls Body Fat Mass, Associated With Serum Zonulin, in Overweight and Obese Adults—Randomized Controlled Trial. EBioMedicine.

[52] Do H.P., Tran B.X., Nguyen C.T., Van Vo T., Baker P.R.A., Dunne M.P. (2019). Inter-partner violence during pregnancy, maternal mental health and birth outcomes in Vietnam: A systematic review. Child. Youth Serv. Rev..

[53] Gishti O., Gaillard R., Durmus B., Abrahamse M., Van Der Beek E.M., Hofman A., Franco O.H., De Jonge L.L., Jaddoe V.W.V. (2015). BMI, total and abdominal fat distribution, and cardiovascular risk factors in school-age children. Pediatr. Res..

[54] Hsieh C.-J., Wang P.-W., Chen T.-Y. (2014). The relationship between regional abdominal fat distribution and both insulin resistance and subclinical chronic inflammation in non-diabetic adults. Diabetol. Metab. Syndr..

[55] Jung S.-P., Lee K.-M., Kang J.-H., Yun S.-I., Park H.-O., Moon Y., Kim J.-Y. (2013). Effect ofLactobacillus gasseriBNR17 on Overweight and Obese Adults: A Randomized, Double-Blind Clinical Trial. Korean J. Fam. Med..

[56] Park D.-Y., Ahn Y.-T., Park S.-H., Huh C.-S., Yoo S.-R., Yu R., Sung M.-K., McGregor R.A., Choi M.-S. (2013). Supplementation of Lactobacillus curvatus HY7601 and Lactobacillus plantarum KY1032 in Diet-Induced Obese Mice Is Associated with Gut Microbial Changes and Reduction in Obesity. PLoS ONE.

[57] Chang B.J., Park S.U., Jang Y.S., Ko S.H., Joo N.M., Kim S.I., Kim C.-H., Chang D.K. (2011). Effect of functional yogurt NY-YP901 in improving the trait of metabolic syndrome. Eur. J. Clin. Nutr..

[58] Drissi F., Merhej V., Angelakis E., Kaoutari A.E., Carrière F., Henrissat B., Raoult D. (2014). Comparative genomics analysis of Lactobacillus species associated with weight gain or weight protection. Nutr. Diabetes.

[59] Zarrati M., Shidfar F., Nourijelyani K., Mofid V., Hosseinzadeh-Attar M.J., Bidad K., Najafi F., Gheflati Z., Chamari M., Salehi E. (2013). Lactobacillus acidophilus La5, Bifidobacterium BB12, and Lactobacillus casei DN001 modulate gene expression of subset specific transcription factors and cytokines in peripheral blood singlenuclear cells of obese and overweight people. BioFactors.

[60] Kelishadi R., Farajian S., Safavi M., Mirlohi M., Hashemipour M. (2014). A randomized triple-masked controlled trial on the effects of synbiotics on inflammation markers in overweight children. J. Pediatr..

[61] De Oliveira M.N., Sivieri K., Alegro J.H.A., Saad S.M.I. (2002). Aspectos tecnológicos de alimentos funcionais contendo probióticos. Rev. Bras. Ciênc. Farm..

[62] Jensen M.D., Ryan D.H., Apovian C.M., Ard J.D., Comuzzie A.G., Donato K.A., Hu F.B., Hubbard V.S., Jakicic J.M., Kushner R.F. (2014). 2013 AHA/ACC/TOS Guideline for the Management of Overweight and Obesity in Adults: A report of the American college of cardiology/American heart association task force on practice guidelines and the obesity society. J. Am. Coll. Cardiol..

[63] Mazloom K., Siddiqi I., Covasa M. (2019). Probiotics: How Effective Are They in the Fight against Obesity?. Nutrients.

[64] Cerdó T., García-Santos J.A., Bermúdez M.G., Campoy C. (2019). The Role of Probiotics and Prebiotics in the Prevention and Treatment of Obesity. Nutrients.

[65] Benahmed A.G., Gasmi A., Doşa A., Chirumbolo S., Mujawdiya P.K., Aaseth J., Dadar M., Bjørklund G. (2021). Association between the gut and oral microbiome with obesity. Anaerobe.

[66] Radaic A., Kapila Y.L. (2021). The oralome and its dysbiosis: New insights into oral microbiome-host interactions. Comput. Struct. Biotechnol. J..

[67] de Simone C. (2019). The Unregulated Probiotic Market. Clin. Gastroenterol. Hepatol..

