# Supplementary material for: Effects of Probiotics and Synbiotics on Weight Loss in Subjects with Overweight or Obesity: A Systematic Review
Source: Nutrients. 2021 Oct 17;13(10):3627. doi: 10.3390/nu13103627 (PMC8540110; doi:10.3390/nu13103627)
Supplement: Supplementary file 1 [file nutrients-13-03627-s001.zip › nutrients-1380436-supplementary.pdf]

## Supplementary Material:

**Table S1.** Search strategies used for this systematic review.

| Search strategy | Search filters | Articles found |
|---|---|---|
| | Pubmed | |
| ((probiotics [MeSH terms]) OR (Lactobacillus [MeSH terms]) OR (Synbiotics [MeSH terms] )) AND ((overweight [MeSH Terms]) OR (obesity [MeSH Terms]) OR (Weight Loss [MeSH Terms])) | Published in the last 10 years<br>Article Type: Clinical Trials<br>Species: Humans | 101 |
| | Scopus | |
| TITLE-ABS (probiotic* OR prebiotic* OR synbiotic* OR lactobacillus OR bifidobacteria) AND TITLE-ABS ( overweight OR obesity OR "weight loss") AND NOT mice | Published in the last 10 years<br>Document type: Article | 47 |
| | Web of Science | |
| (TS=(probiotic* OR prebiotic* OR synbiotic* OR lactobacillus OR bifidobacteria) AND TS=(overweight OR obesity OR "weight loss") AND TI= (trial) NOT TS= (mice OR mouse OR rat OR animal*) AND PY=(2011-2021)) | Field Tags: TS | 56 |

ABS: Abstract; TS: Topic; TI: Title

**Table S2.** Assessment of the methodological quality of the included clinical trials, using the Jadad scale.

| Author [ref] | Is the item described randomized? (*) | Is the study describedas double blind? (*) | Are dropouts & exclusions from that study described? (*) | Is the method of randomization appropriate? (**) | Is the double blind method appropriate? (**) | Final score |
|---|---|---|---|---|---|---|
| Gomes et al. [35] | 1 | 1 | 1 | 1 | 1 | 5 |
| Gutiérrez-Repiso *et al.* [42] | 1 | 0 | 1 | 1 | -1 | 2 |
| Hadi *et al.* [41] | 1 | 1 | 1 | 1 | 1 | 5 |
| Higashikawa *et al.* [34] | 1 | 1 | 1 | 1 | 1 | 5 |
| Ipar *et al.* [31] | 1 | 0 | 1 | -1 | -1 | 0 |
| Jung *et al.* [30] | 1 | 1 | 1 | 1 | 1 | 5 |
| Kadooka *et al.* [27] | 1 | 1 | 0 | 1 | 1 | 4 |
| Kadooka *et al.* [23] | 1 | 1 | 0 | 1 | 1 | 4 |
| Kianifar *et al.* [37] | 1 | 1 | 1 | -1 | -1 | 1 |
| Kim *et al.* [38] | 1 | 1 | 1 | 1 | 1 | 5 |
| Lee *et al.* [29] | 1 | 1 | 1 | -1 | 1 | 4 |
| Lim *et al.* [46] | 1 | 1 | 1 | -1 | -1 | 1 |
| Madjd *et al.* [33] | 1 | 0 | 1 | -1 | -1 | 0 |
| Michael *et al.* [43] | 1 | 1 | 1 | 1 | 1 | 5 |
| Michael *et al.* [48] | 1 | 1 | 1 | 1 | 1 | 5 |
| Mohammadi-Sartang *et al.* [36] | 1 | 1 | 1 | -1 | 1 | 3 |
| Omar et al. [24] | 0 | 1 | 0 | 1 | 1 | 3 |
| Pedret *et al.* [39] | 1 | 1 | 1 | 1 | 1 | 5 |
| Rahayu *et al.* [49] | 1 | 1 | 1 | 1 | 1 | 5 |
| Razmpoosh *et al.* [44] | 1 | 0 | 1 | 1 | -1 | 2 |
| Safavi *et al.* [26] | 1 | 1 | 1 | -1 | 1 | 3 |
| Sanchez *et al.* [25] | 1 | 1 | 1 | 1 | 1 | 5 |
| Sergeev *et al.* [47] | 0 | 0 | 0 | -1 | -1 | -2 |
| Song *et al.* [45] | 1 | 1 | 1 | 1 | 1 | 5 |
| Stenman *et al.* [32] | 1 | 1 | 1 | 1 | 1 | 5 |
| Sudha *et al.* [40] | 1 | 1 | 1 | 1 | 1 | 5 |
| Zarrati *et al.* [28] | 1 | 1 | 0 | 1 | 1 | 4 |

(*) Yes = 1 /No = 0

(**) Yes = 1 / No = −1.
